# The Difference

**Published:** 1876-12

**Authors:** 


### *THE DIFFERENCE.*

The difference between being shut up in a stifling and offensive sleeping car all night, and enjoying a well-warmed and well-ventilated state-room n a steamer, has lately presented itself to us most forcibly. We went to Boston one night by rail and came back by the boat of the Fall River line—the steamer "Providence." The night on the cars was passed in a berth of a palace car, and there was not an hour after we crawled into our berth until we crawled out again next morning that we were not profoundly impressed with two thoughts,—first, that we were being poisoned by bad air and dust, and second, that we were being shaken and jolted until we were bruized and sore. We finally awoke with a severe headache which did not wholly pass away until 24 hours later. We were conscious that we had been poisoned by breathing an atmosphere very close and bad and unwholesome, and greatly vitiated by passing in and out of many lungs. We felt that we had committed an error, and our repentance was unquestionable, thorough, and sincere.

But we behaved more rationally on our return. We secured a good stateroom on the steamer alluded to, and, leaving Boston at 5½ P. M., we took possession of it at about 7 o'clock at Fall River. It was a cold night —only a day or two after "Thanksgiving," and we obtained a good supply of blankets from the attendant. We opened our window, for ventilation, and went to sleep. We awoke in New York at 7 o'clock next morning feeling entirely fresh and elastic, as might be expected after a night of perfect repose in a well ventilated room, without jar or noise or discomfort. We are not stockholders in either line. We speak only as one having some little authority in the matter of hygiene when we say that it is our duty at all times to endeavor to provide for ourselves good air and wholesome surroundings. It is safe to put one's trust in "Providence."

